# Factors associated with creatine kinase variability among statin users in routine outpatient care: a retrospective cross-sectional study

**DOI:** 10.3389/fcvm.2026.1903467

**Published:** 2026-08-28

**Authors:** Aymen Alqurain, Ahmed K. Alsultan, Mariam K. Alamoudi, Maram Almohaywi, Afnan Shakoori, Hassan Al Gharash, Nida Alsaffar, Amani S. Alrossies

**Affiliations:** 1 Department of Clinical Practice, Faculty of Pharmacy, Northern Border University, Rafha, Saudi Arabia; 2 Al-Dawaa Medical Services Company, Eastern Province Alhassa, Alhafouf, Saudi Arabia; 3 Department of Pharmacology, College of Pharmacy, Prince Sattam Bin Abdulaziz University, Al-Kharj, Saudi Arabia; 4 Saudi Food and Drug Authority, Riyadh, Saudi Arabia; 5 Department of Clinical Laboratory Sciences, Faculty of Applied Medical Sciences, Umm Al-Qura University, Makkah, Kingdome of Saudi Arabia; 6 Emergency and Intensive Care Department, College of Nursing, Northern Border University, Arar, Saudi Arabia; 7 Independent Researcher, Dammam, Saudi Arabia; 8 Department of Pharmacy Practice, College of Pharmacy, Princess Nourah Bint Abdulrahman University, Riyadh, Saudi Arabia

**Keywords:** creatine kinase, drug safety, risk factors, statin, statin-associated muscle symptoms

## Abstract

**Background:**

Statin therapy is frequently associated with concerns regarding creatine kinase (CK) elevation and potential muscle toxicity. Although clinically significant statin-associated muscle injury is uncommon, the interpretation of routine CK variability during outpatient statin therapy remains uncertain.

**Objectives:**

To evaluate CK variability among outpatient statin users and identify patient-and treatment-related factors associated with CK levels.

**Methods:**

A retrospective cross-sectional study was conducted among adult outpatient attending cardiology, endocrinology, or internal medicine clinics at a tertiary care center in Saudi Arabia between January-2020 to December-2021. Demographic characteristics, comorbidities, statin exposure, statin intensity, concomitant medications, renal function, and laboratory parameters were extracted from electronic medical records. Multivariable linear regression analyses were performed to identify factors independently associated with CK levels.

**Results:**

A total of 6,517 patients were included, of whom 3,223 (49.5%) received statin therapy. Statin users had higher CK levels than non-users (112 vs. 80 IU/L, *p* < 0.001). CK values were generally distributed within a narrow range, with a median of 94 IU/L (IQR 68–121) and 95% of measurements below 157 IU/L. In the fully adjusted model, statin use (*β* = 31.84, 95% CI 29.98–33.69; *p* < 0.001) and male sex (*β* = 13.95, 95% CI 12.36–15.53; *p* < 0.001) were independently associated with higher CK levels. Thyroid disorder demonstrated a modest association with CK levels (*β* = 2.60, 95% CI 0.32–4.88; *p* = 0.026).

**Conclusion:**

Statin use and male sex were the primary factors associated with CK variability in this outpatient population. Despite modestly higher CK concentrations among statin users, most measurements remained within a relatively narrow range, and no dose-response relationship was observed according to statin intensity or statin type. These findings provide real-world evidence to support the interpretation of routine CK measurements during outpatient statin therapy.

## 1 Introduction

Statins are among the most widely prescribed medications worldwide and remain the cornerstone of lipid-lowering therapy for the primary and secondary prevention of atherosclerotic cardiovascular disease (1, 2). Their efficacy in reducing low-density lipoprotein cholesterol (LDL-C) levels and lowering the risk of major cardiovascular events has been consistently demonstrated across diverse patient populations (1, 3). Consequently, long-term statin therapy is recommended by international guidelines and is frequently prescribed in routine clinical practice. As statin use continues to increase, ongoing evaluation of their safety profile remains essential to support optimal therapeutic outcomes and maintain patient adherence (4).

Although statins are generally well tolerated, concerns regarding statin-associated muscle symptoms (SAMS) remain one of the most common reasons for treatment discontinuation or reduced adherence (4–6). Creatine kinase (CK) is frequently used as a biochemical marker when evaluating potential muscle-related adverse effects during statin therapy (7–10). However, CK concentrations can vary substantially among individuals and may be influenced by several physiological and clinical factors, including sex, age, body composition, physical activity, renal function, and comorbid conditions. Importantly, isolated increases in CK do not necessarily indicate clinically significant muscle injury, particularly when values remain within or near the normal laboratory range and occur in otherwise asymptomatic individuals (11).

Previous studies examining CK during statin therapy have primarily focused on symptomatic patients, severe CK elevations, or rare muscle-related adverse events such as myopathy and rhabdomyolysis (1, 2, 5, 12). In contrast, relatively little attention has been given to understanding routine CK variability among stable outpatient populations receiving statin therapy. Consequently, the factors that contribute to differences in CK measurements observed during everyday clinical practice remain incompletely characterised. Improved understanding of these factors may facilitate more accurate interpretation of CK concentrations and help distinguish expected biological variability from clinically meaningful abnormalities.

Despite considerable research that has been conducted on SAMS, the understanding of moderate CK fluctuations observed during standard outpatient monitoring is still inadequately defined. Most prior research focused on symptomatic individuals, clinically significant elevations in CK levels, or statin intolerance, but physicians frequently encounter asymptomatic patients with minor CK variations during routine follow-up. Therefore, this study aimed to identify factors associated with CK variability in a large outpatient cohort receiving routine statin therapy and to provide evidence to support the interpretation of CK measurements encountered in everyday practice.

## 2 Methodology

### 2.1 Study design and study population

A retrospective cross-sectional study was conducted using electronic medical records from adult outpatients attending cardiology, endocrinology, and internal medicine clinics at Al-Qatif Central Hospital (QCH), a tertiary care centre, in the Eastern Region of Saudi Arabia. Data were collected for encounters occurring between 1 January 2020 and 31 December 2021.

Adult patients aged 40 years or older with at least one recorded CK measurement during the study period were eligible for inclusion. The age of 40 years was set as the minimum age threshold due to the epidemiological rise in cardiometabolic diseases and statin eligibility beginning at this age group (13). Patients were excluded if they were under 40 years of age; visited outpatient clinics outside cardiology, endocrinology, or internal medicine specialties; or had incomplete demographic, prescription, or laboratory records relevant to the study outcomes. For patients with several visits during the study period, only the initial visit was included to minimize within-patient clustering and overrepresentation. This approach was implemented to establish a representative outpatient cohort while reducing selection bias associated with insufficient clinical or laboratory documentation.

Patients were classified according to statin exposure status based on active prescriptions recorded within the electronic medical record. Individuals receiving atorvastatin, rosuvastatin, simvastatin, or other statins were classified as statin users, whereas patients without an active statin prescription served as the comparison group. Where multiple CK measurements were available, the CK value corresponding to the study encounter was used for analysis.

This study was reported in compliance with the Strengthening the Reporting of Observational Studies in Epidemiology (STROBE) standards for cross-sectional research (14). No preliminary sample size calculation was conducted, as this was a retrospective observational study. All eligible patients who satisfied the inclusion criteria during the study period were included to maximize representativeness, statistical precision, and reflection of real-world outpatient prescribing patterns. The overall proportion of missing data for variables in multivariable analyses was minimal, and no imputation was conducted. All analyses were performed using a complete-case approach.

Several strategies were implemented to minimize potential bias. Selection bias was mitigated by including all eligible outpatient visits within the specified study period and clinical specialties. Information bias was mitigated by employing standardized electronic medical records, pharmacy databases, and laboratory systems. To mitigate misclassification, statin exposure was defined by active prescriptions recorded at the index visit, while CK levels were obtained from routine laboratory measurements conducted as part of standard clinical care.

### 2.2 Data collection, measures, and definitions

#### 2.2.1 Prescribed medication, statin exposure, and dose intensity

Prescribed medications were classified using the Anatomical Therapeutic Chemical (ATC) classification system (15). The total number of prescribed medications (NPM) was calculated and recorded. Statin dose intensity was classified according to established guideline-based definitions consistent with lipid management guideline recommendations (16, 17). Dose intensity was chosen as the primary exposure variable to represent actual prescribing practices and the comparative potency of statins rather than focusing solely on individual statin agents (17).

#### 2.2.2 Covariates and confounders

Demographic covariates included age, sex, and body weight were collected due to their established impact on statin pharmacokinetics, muscle mass, and baseline CK levels. Comorbid conditions were identified from electronic medical records and supplemented using the Rx-Risk Comorbidity Index to improve the identification of chronic conditions based on medication prescribing patterns (18). All diagnoses were subsequently coded using the International Classification of Diseases, version 10 (ICD-10) (19). Rheumatoid arthritis and osteoarthritis were categorized as “arthritis-related diseases” due to diagnostic overlap, whereas heart failure, hypertension and ischemic heart diseases were grouped as cardiovascular diseases. The Charlson Comorbidity Index (CCI) was CCI was calculated to describe overall comorbidity burden and clinical complexity (18, 20, 21).

Renal function was assessed using creatinine clearance (CrCl), calculated via the Cockcroft-Gault equation, which remains widely used for dose adjustment in older adults (22). The serum creatinine levels used for determining CrCl were obtained from laboratory tests that were performed either at the same outpatient visit as the CK measurement or within a maximum of seven days as possible. When multiple creatinine measurements were available during this period, the result that was closest to the CK test date was chosen.

### 2.3 Statistical analysis

Statistical analyses were performed using IBM SPSS Statistics (version 26). Continuous variables were summarized using means with standard deviations or medians with interquartile ranges, as appropriate, while categorical variables were reported as frequencies and percentages. The Shapiro–Wilk test was used to evaluate the normality of continuous variables. To characterize the distribution of CK measurements within the study population, percentile distributions and quartile-based descriptive analyses were additionally performed.

Descriptive univariate comparisons between statin users and non-users were performed using independent t-tests or Mann–Whitney U tests for continuous variables, and chi-square or Fisher's exact tests for categorical variables, as appropriate. Comparisons among statin dose-intensity groups were performed using ANOVA or Kruskal–Wallis tests, as appropriate.

Exploratory subgroup analyses were performed to compare statin prescribing and CK levels within clinically relevant patient populations. Separate univariate analyses were conducted for patients with and without diabetes mellitus, anemia, and cardiovascular disease.

Multivariable linear regression models were developed among statin users to identify factors independently associated with CK levels. Sequential models were developed to evaluate the influence of demographic characteristics, statin exposure, and clinical variables. Covariates were selected *a priori* based on biological plausibility, previously published literature, and clinical relevance. The fully adjusted model included age, sex, body weight, CrCl, diabetes mellitus, hypertension, ischemic heart disease, heart failure, thyroid disorder, renal disease, and liver disease. Additional analyses restricted to statin users were performed to examine the independent associations of statin intensity and statin type with CK levels.

Model assumptions, including linearity, homoscedasticity, residual normality, and absence of multicollinearity, were evaluated prior to interpretation. Results are presented as unstandardized coefficients (β) with corresponding 95% confidence interval (95% CI) and *p*-value. A two-sided *p*-value < 0.05 was considered statistically significant.

### 2.4 Ethical approvals

The data used in this study were collected as per the approval from the Institutional Review Boards (IRB) of both Mohammed Al-Mana College for Medical Sciences (Approval No. SR/RP/79; 17 February 2022) and QCH (Approval No. QCH-SRECO 19-2022; 8 June 2022). Informed consent was waived due to the retrospective nature of data collection, and complete de-identification of patient information was applied.

## 3 Results

### 3.1 Baseline characteristics

A total of 6,517 patients met the inclusion criteria. The median age was 52 years (45–63), and 52% were female (Table 1). The median CCI score was 2 (1–3), the median NPM was 5 (2–10), the mean CrCl was 96 mL/min (± 46), and the median body weight was 70 kg (63–80) (Table 1).

**Table 1:** Baseline characteristics of patients classified based on statin prescription.

Characteristics	Entire cohort	Statin users	Statin non-users	*p*-value
	*n* *=* *6,517*	*n* *=* *3,223*	*n* *=* *3,294*	
Age (year), median (IQR)	52 (45–63)	52 (45–62)	52 (45–63)	0.2
Gender (Female sex), *n* (%)	3,380 (52)	1,682 (52)	1,698 (52)	0.6
CCI, median (IQR)	2 (1–3)	2 (1–3)	2 (1–3)	0.8
NPM, median (IQR)	5 (2–10)	5 (3–10)	4 (2–9)	< 0.001
CrCl (mL/min), mean (SD)	96 (46)	92 (46)	101 (45)	< 0.001
Body Weight (Kg), median (IQR)	70 (63–80)	70 (62–80)	70 (65–80)	0.2
Comorbidities				
Diabetes mellitus, *n* (%)	2,275 (35)	1,798 (56)	477 (15)	< 0.001
Liver disease, *n* (%)	340 (5)	223 (7)	117 (4)	< 0.001
Renal disease, *n* (%)	205 (3)	118 (4)	87 (3)	0.02
Anemia, *n* (%)	2,163 (33)	998 (31)	1,165 (35)	< 0.001
Heart Failure, *n* (%)	3,072 (47)	2,235 (69)	837 (25)	< 0.001
Hypertension, *n* (%)	3,168 (39)	2,302 (71)	866 (26)	< 0.001
Ischemic Heart Disease, *n* (%)	3,503 (54)	2,485 (77)	1,018 (31)	< 0.001
Thyroid disorder, *n* (%)	839 (13)	429 (13)	410 (12)	0.3

IQR, interquartile range; SD, standard deviation; n, number of participants; CCI, Charlson comorbidity index; NPM, number of prescribed medications; CrCl, creatinine clearance.

Statins were prescribed to 50% of the included patients. Compared with non-users, statin users had a higher median NPM (5 vs. 4, *p* < 0.001) and lower CrCl values (92 vs. 101 mL/min, *p* < 0.001) (Table 1). Compared with non-users, statin users had a greater frequency of diabetes mellitus (56% vs. 15%, *p* < 0.001), heart failure (69% vs. 25%, *p* < 0.001), hypertension (71% vs. 26%, *p* < 0.001), and ischemic heart disease (77% vs. 31%, *p* < 0.001) (Table 1). Anemia was less prevalent among statin users than non-users (31% vs. 35%, *p* < 0.001) (Table 1).

### 3.2 Statin prescribing patterns

**Figure 1:**
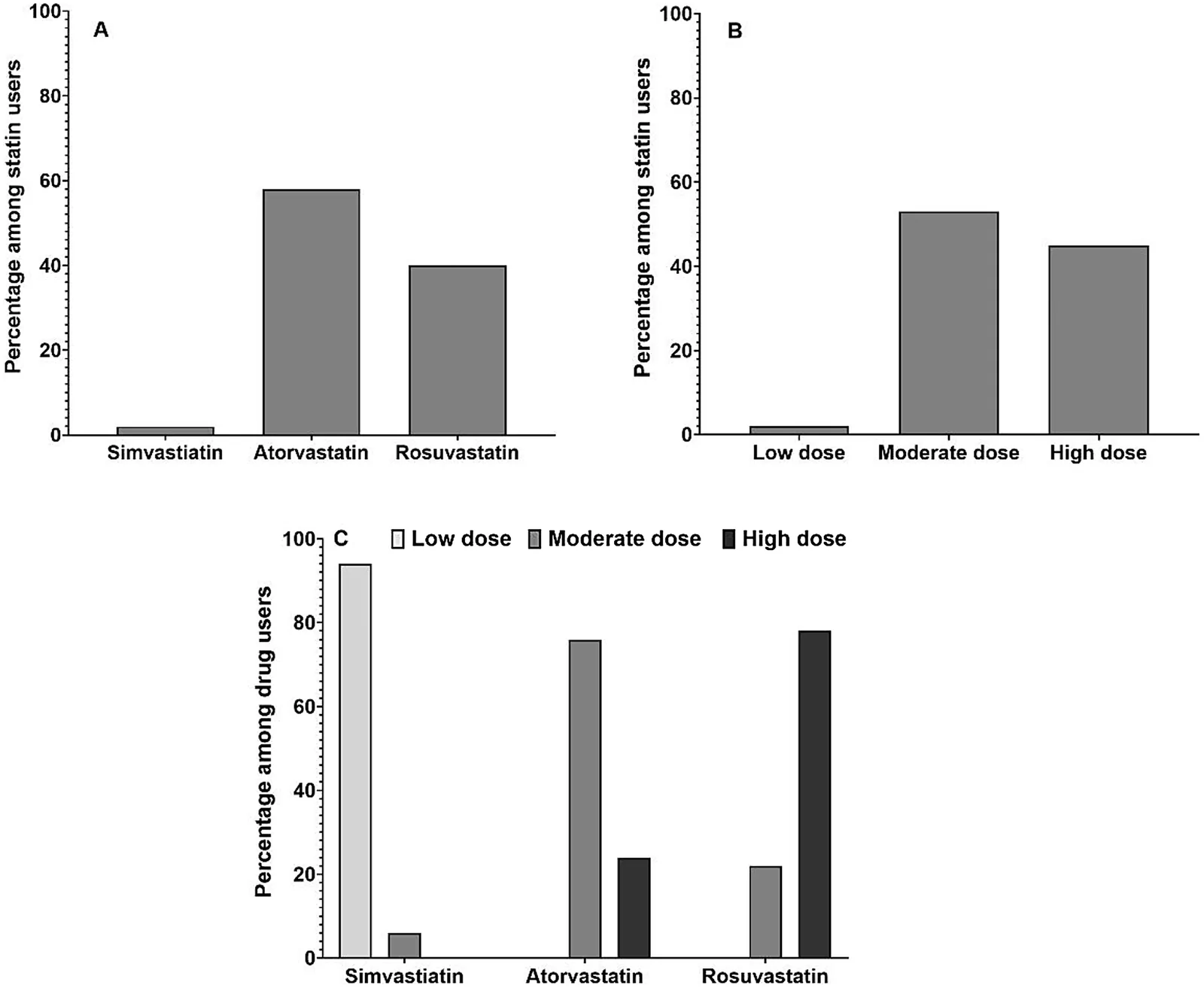
Patterns and prevalence of statin prescriptions. **(Panel A)** presents a pattern of statin prescription based on the selected drug. **(Panel B)** presents a pattern of statin prescription based on dose intensity. **(Panel C)** presents the pattern of statin prescription for each agent based on dose intensity.

Atorvastatin (59%) and rosuvastatin (40%) were the most commonly prescribed statins (Figure 1). Moderate-intensity statin therapy accounted for 53% of prescriptions, followed by high-intensity therapy (45%), while low-intensity statin therapy was uncommon (2%) and observed exclusively with simvastatin (Figure 1).

### 3.3 Creatine kinase distribution among the cohort

CK concentrations were generally distributed within a relatively narrow range across the study cohort. The median CK level was 94 IU/L (IQR 68–121), with 95% of values below 157 IU/L and 99% below 175 IU/L (Supplementary Table S1). The proportion of statin users increased progressively across CK quartiles, ranging from 23.1% in the lowest quartile to 82.6% in the highest quartile. A statistically significant association was observed between CK quartile membership and statin exposure (χ^2^ = 1,186.5, df = 3, *p* < 0.001), indicating a strong gradient in statin use across increasing CK levels. Similarly, the proportion of male patients increased from 36.7% in the lowest CK quartile to 62.4% in the highest quartile. In contrast, age, medication burden, and comorbidity burden demonstrated relatively limited variation across CK quartiles (Supplementary Table S2).

**Figure 2:**
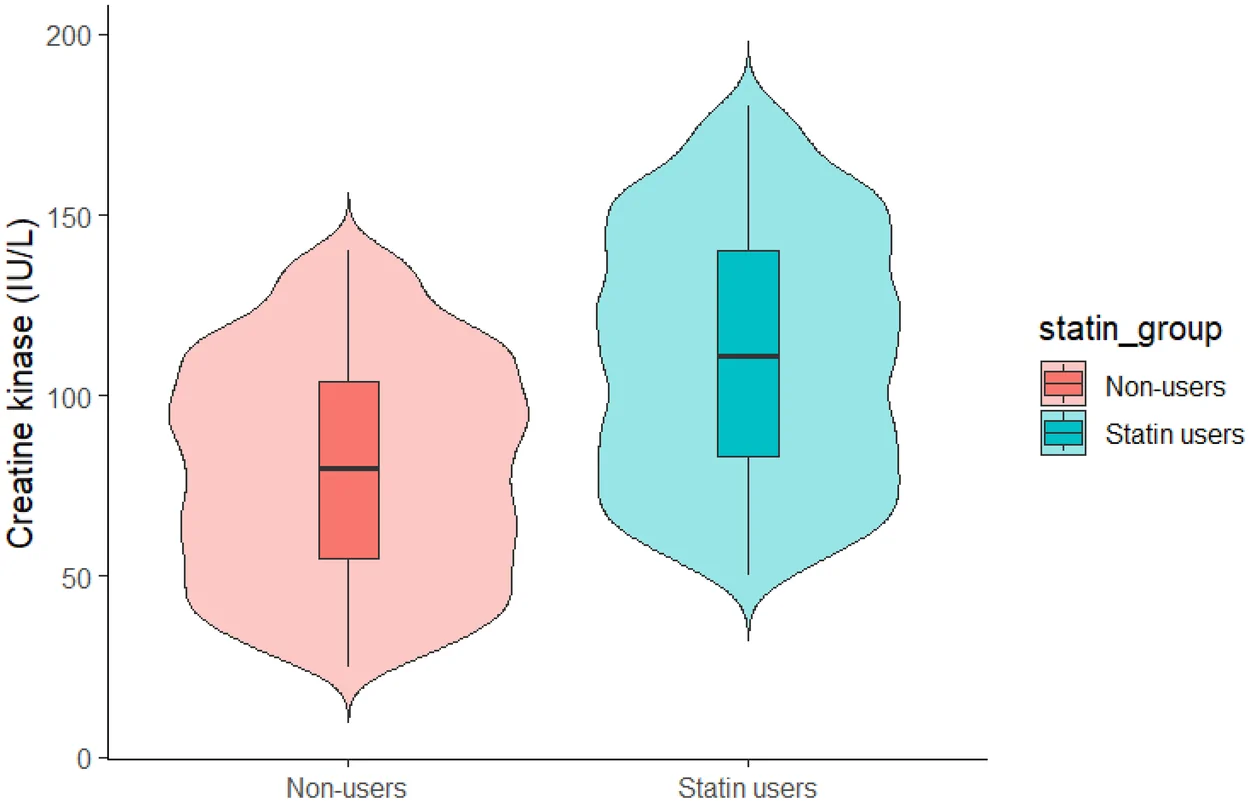
Distribution of creatine kinase levels according to statin exposure status.

Figure 2 demonstrates the distribution of CK measurements according to statin exposure status. Although statin users exhibited modestly higher CK levels than non-users, substantial overlap between the distributions was observed, indicating that most CK measurements remained within routine outpatient ranges regardless of statin exposure.

### 3.4 Statin exposure and creatine kinase level

Statin users had modestly higher mean creatine kinase (CK) levels compared with non-users (112 vs. 80 IU/L, *p* < 0.001), with an absolute difference between groups was approximately 32 IU/L. (Supplementary Material Table S1). In unadjusted analyses, no statistically significant differences in CK levels were observed across statin dose-intensity categories.

Further analyses revealed that average Ck level was slightly higher among patients with diabetes mellitus (104 vs. 91 IU/L, *p* < 0.001) and those with cardiovascular disease (102 vs. 87 IU/L, *p* < 0.001), but moderately lower among patients with anemia (94 vs. 96 IU/L, *p* = 0.01) compared to those with no documented disease status. Interestingly, statin prescribing was higher among diabetic patients (79% vs. 34%, *p* < 0.001), cardiovascular disease patients (69% vs. 23%, *p* < 0.001), but less often among patients with anemia (46% vs. 51, *p* < 0.001) compared to those with no disease status (Supplementary Materials Tables S3–S5).

### 3.5 Characteristics by dose intensity

**Figure 3:**
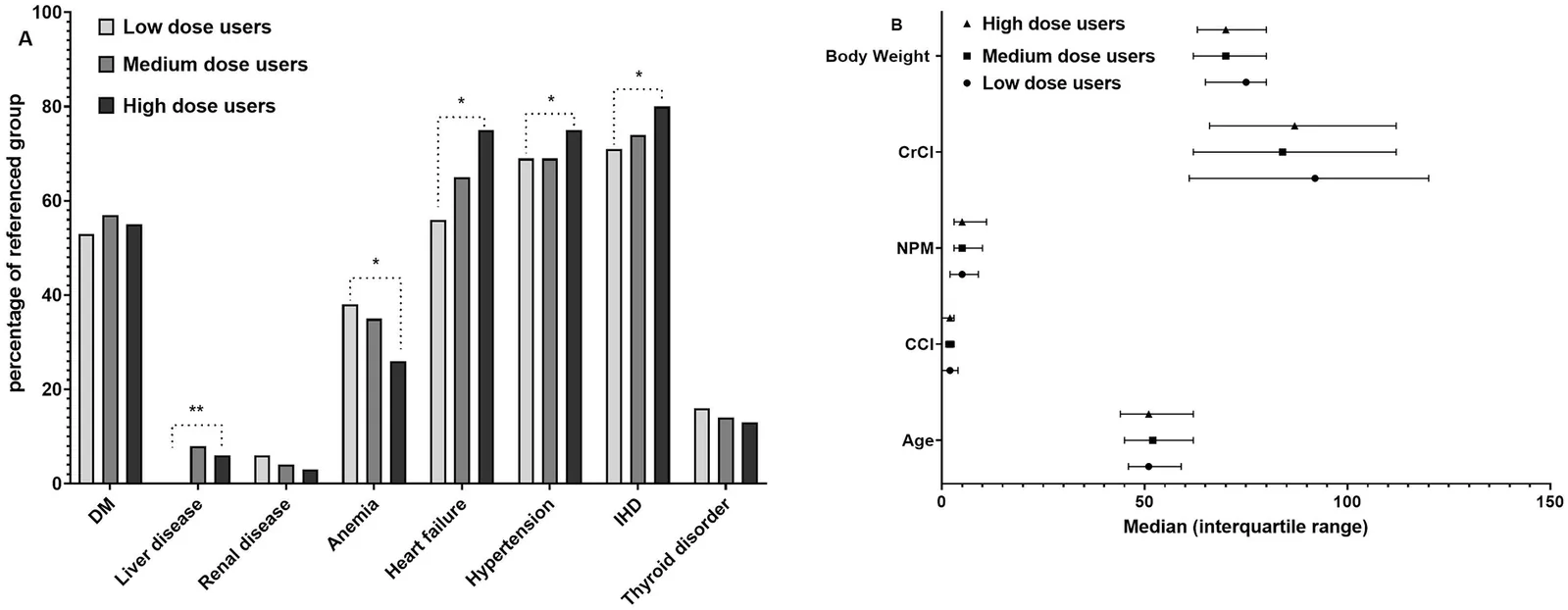
Characteristics of statin users classified based on the intensity of statin dosage prescribed (Panel **A** presents differnce in comorbidites, Panel **B** presents difference in baseline characteristcs). DM, diabetes mellitus; IHD, ischemic heart disease; CCI, Charlson comorbidity index; NPM, number of prescribed medications; CrCl, creatinine clearance*.*

Patients prescribed high-intensity statins had a higher prevalence of heart failure (75% vs. 65% vs. 56%, *p* < 0.001), hypertension (75% vs. 69% vs. 69%, *p* < 0.001), and ischemic heart disease (80% vs. 74% vs. 71%, *p* < 0.001) compared with those receiving moderate- or low-intensity statins (Figure 3). Anemia was less prevalent among patients prescribed high-intensity statins compared with those receiving moderate- or low-intensity statins (26% vs. 35% vs. 38%, *p* < 0.001) (Figure 3). Liver disease was more prevalent among moderate-intensity users (8%) (Figure 3).

### 3.6 Characteristics by sex among statin users

**Figure 4:**
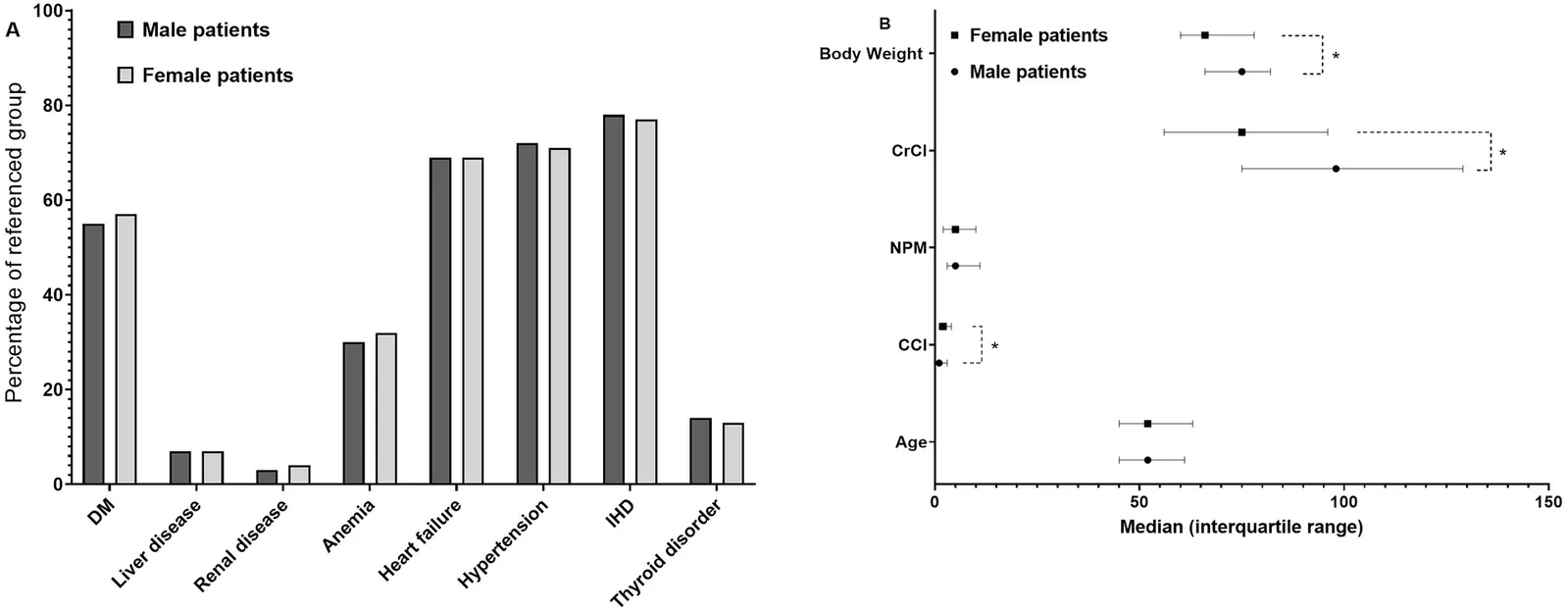
Characteristics of statin users classified based on gender differences. (Panel **A** presents differnce in comorbidites, Panel **B** presents difference in baseline characteristcs). DM, diabetes mellites; IHD, ischemic heart disease; CCI, Charlson comorbidity index; NPM, number of prescribed medications; CrCl, creatinine clearance.

Among statin users, male patients had a higher average CrCl (106 vs. 80 mL/min, *p* < 0.001) and higher median body weight (75 vs. 66 kg, *p* < 0.001), but lower median CCI score (1 vs. 2, *p* < 0.001) compared with female patients (Figure 4).

### 3.7 Multivariable linear regression analysis

**Figure 5:**
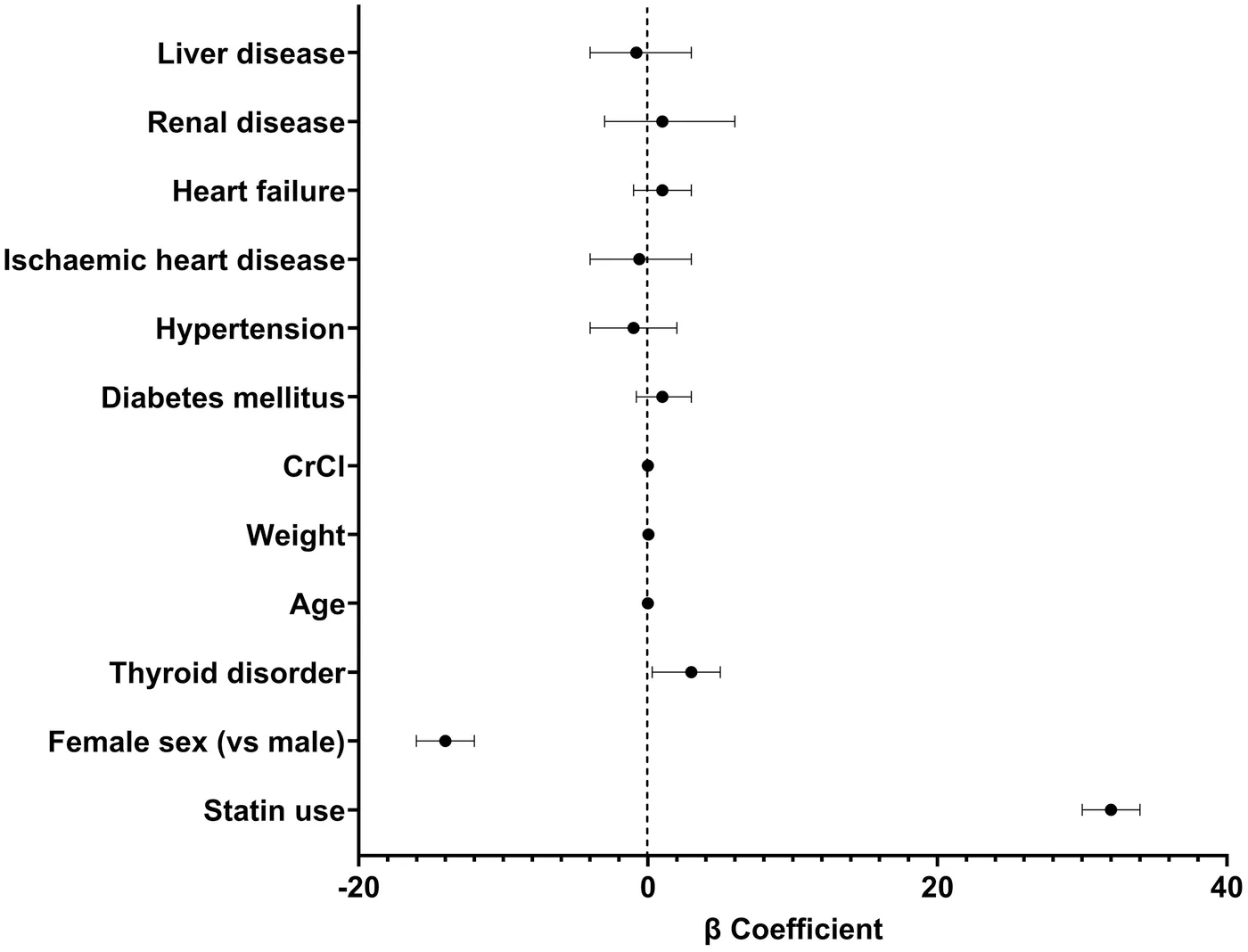
Factors associated with creatine kinase levels in the fully adjusted model. *β* coefficients represent adjusted differences in CK levels (IU/L) derived from multivariable linear regression analysis. Confidence intervals (95% CI) are presented for all regression coefficients. Positive coefficients indicate higher CK levels, whereas negative coefficients indicate lower CK levels relative to the reference group. CrCl, creatine clearance.

In the fully adjusted multivariable model, statin use remained independently associated with modestly higher CK levels (*β* = 31.84, 95% CI 29.98 to 33.69; *p* < 0.001). Female sex was independently associated with moderately lower CK levels than male sex (*β* = −13.95, 95% CI −15.53 to −12.36; *p* < 0.001), while thyroid disorder demonstrated a modest positive association with modestly higher CK levels (*β* = 2.60, 95% CI 0.32 to 4.88; *p* = 0.026) (Figure 5).

## 4 Discussion

This study evaluated CK variability among more than 6,500 adult outpatients and identified several factors associated with CK levels during routine clinical care. Rather than evaluating clinically overt SAMS, the present study focused on understanding routine CK variability encountered during outpatient statin therapy, thereby addressing a common clinical scenario in which isolated CK elevations require interpretation in the absence of clear muscle symptoms. Statin users exhibited modestly higher CK levels than non-users, and statin exposure remained independently associated with CK variability after adjustment for demographic and clinical characteristics. Male sex was the strongest patient-related factor associated with CK levels variability, whereas statin intensity and statin type were not independently associated with CK concentrations among statin users. Heart failure and diabetes mellitus were prevalent among the cohort and patients presented with these chronic diseases had a modestly higher average CK level. Importantly, most CK measurements remained within a relatively narrow range, suggesting that routine CK variability may be more common than clinically significant muscle injury in outpatient populations receiving statin therapy. Collectively, these findings suggest that routine CK variability in outpatient statin users is primarily influenced by patient characteristics and statin exposure rather than statin intensity or statin type, providing a practical framework for interpreting CK measurements in everyday clinical practice.

In this outpatient cohort, approximately half of the cohort was prescribed statins, while atorvastatin and rosuvastatin were the most commonly prescribed statins, with the majority of patients receiving moderate or high intensity therapy. This prescribing pattern is consistent with recent lipid management guideline recommendations that prioritize these medications for patients with high cardiovascular risk (4, 23). The high prevalence of moderate to high intensity statin use observed in this study (53% and 45%, respectively) reflects appropriate risk-based prescribing rather than aggressive or non-standard practice. Interestingly, this ratio exceeds rates reported in prior local studies, where high-intensity prescribing among diabetic patients ranged between 11% and 15% (24, 25). Although higher-intensity statins were more commonly prescribed to patients with greater comorbidity burden, statin dose intensity was not independently associated with CK levels after multivariable adjustment.

The absence of an independent association between statin intensity and CK concentrations is an important observation of the current study. Although concerns regarding muscle toxicity often increase with higher statin doses, routine CK variability in this outpatient cohort did not differ according to statin intensity after adjustment for demographic and clinical factors. Given the cross-sectional design, this finding should not be interpreted as evidence against a dose-related effect, as residual confounding and other unmeasured factors cannot be excluded. Nevertheless, the observed pattern is consistent with current guideline recommendations that CK measurements should be interpreted alongside the patient's clinical presentation rather than according to statin intensity alone (4). Prospective studies incorporating serial CK measurements and statin-associated muscle symptoms are needed to confirm these observations.

The observed association between statin exposure and modestly higher CK levels is consistent with the established biological effects of statins on skeletal muscle (5, 11). However, the magnitude and distribution of CK values observed in this study suggest that these differences should be interpreted cautiously. Although statin users demonstrated slightly higher CK concentrations than non-users, the majority of measurements remained within a relatively narrow range, with 95% of values below 157 IU/L. These findings support previous observations that modest CK elevations may occur during statin therapy without necessarily indicating clinically meaningful muscle injury (4, 7). Consequently, isolated CK elevations should be interpreted within the broader clinical context, including symptom burden and the presence of alternative causes of CK variability (4). Notably, the crude difference in CK levels between statin users and non-users (approximately 32 IU/L) was nearly identical to the adjusted estimate obtained in multivariable analyses (31.84 IU/L), suggesting minimal residual confounding by measured demographic and clinical factors.

Male sex emerged as the most consistent patient-related factor associated with CK variability across all regression models. This finding is biologically plausible and consistent with previous literature demonstrating modestly higher baseline CK concentrations among men, likely reflecting differences in skeletal muscle mass, muscle turnover, and physiological CK release (26–29). Importantly, the association remained significant after adjustment for statin exposure, renal function, body weight, and comorbidities, suggesting that sex-related physiological differences contribute substantially to routine CK variability.

Thyroid disorder demonstrated a modest association with CK levels in the fully adjusted model. Although the magnitude of this association was small, thyroid dysfunction has previously been linked to alterations in muscle metabolism and CK concentrations (30, 31). Given the relatively limited effect size observed in the present study, this finding should be interpreted cautiously and warrants confirmation in future investigations.

Another interesting finding was that the high prevalence of heart failure in the study cohort, which indicate the significant cardiovascular disease burden among patients receiving long-term statin therapy in routine outpatient care. While heart failure was not independently associated with CK variability in the primary multivariable analysis, this finding must be interpreted within the context of the complex pathophysiology of heart failure. Prior research has shown that chronic heart failure often is accompanied by skeletal muscle dysfunction, mitochondrial irregularities, and diminished muscle mass, all of which may affect muscle metabolism independently of statin use (32, 33). These findings support our decision to assess cardiovascular disease as a composite subgroup instead of analysing heart failure in isolation, given that cardiovascular comorbidities frequently coexist in patients receiving statins. Future prospective studies that include assessments of heart failure severity, functional status, and serial CK assessments are required in order to clarify the relationship between heart failure and routine CK variability.

Diabetes mellitus was also prevalent among the study cohort, and patients with diabetes mellitus had slightly higher CK levels than those without diabetes mellitus. Diabetes is linked to reduced skeletal muscle metabolism, mitochondrial dysfunction, and diminished muscle quality, potentially affecting CK concentrations irrespective of statin treatment. Prior research has demonstrated that diabetes induces changes in skeletal muscle structure and function, thereby potentially altering muscle biomarker profiles without necessarily signifying clinically relevant muscle damage (34, 35). Considering that diabetes is a major indication for statin therapy in cardiovascular prevention, these findings confirm the applicability of our results to routine clinical practice. Future prospective studies that include glycemic management, diabetes duration, and assessments of muscle function are essential to clarify the relationship between diabetes, statin medication, and CK variability.

This study has several notable strengths. First, to our knowledge, this is among the largest studies evaluating routine CK variability among statin users in outpatient practice and one of the few studies to characterize CK distribution across an unselected real-world population. It includes a large and clinically diverse cohort drawn from a tertiary care hospital, providing a broad representation of statin prescribing patterns within the region. The use of multivariable regression allowed adjustment for key confounding factors, strengthening the validity of the observed associations. Furthermore, the inclusion of CK distribution analyses provided additional insight into the interpretation of routine CK measurements beyond traditional comparisons of mean CK levels. Importantly, this study contributes to the limited literature from the Middle Eastern region on statin prescribing patterns and CK levels, offering valuable insights relevant to clinical practice in Saudi Arabia. This study offers practical insights for clinicians to understand CK variability in a substantial real-world population while concurrently considering demographic, clinical, and treatment-related factors, thereby enhancing the interpretation of CK measurements beyond clinically significant muscle injury.

This study has certain limitations that must be recognized. The study's cross-sectional design limits the ability to determine causality between statin prescribing and CK elevation. Although multivariable regression analyses were performed to adjust for measured confounders, the cross-sectional design precludes causal inference and does not eliminate the possibility of residual confounding or confounding by indication. This study relies on data collected from a single center, potentially constraining its generalizability to wider populations with different demographic and clinical characteristics. However, the large sample size with its diverse characteristics may improve the contextual relevance of the findings to similar Middle Eastern outpatient populations. The outpatient setting of the study diminished the probability of severe acute illness, important clinical data regarding transient illnesses, inflammatory states, or subclinical hypoxic conditions, which could not be incorporated into the regression models.

Residual confounding from acute physiological stressors cannot be completely excluded and may have influenced CK levels in a limited subset of patients. CK levels were measured at a single time point, constraining the assessment of longitudinal variations or fluctuations in CK metabolism. Additionally, CK measurements were obtained during routine clinical care instead of at standardized intervals corresponding to medication dosing, potentially introducing variability in observed concentrations. Despite the concurrent measurement of CrCl and CK levels during the same clinical period, the retrospective nature of the study limited accurate synchronization with the precise timing of statin administration. As a result, transient variations in CrCl or CK associated with recent administration could not be fully characterized. As a cross-sectional study, CK levels were obtained during routine outpatient visits and were not associated with documented acute clinical symptoms, hospitalizations, or hemodynamic instability at the time of testing. Consequently, no direct clinical outcomes linked to elevated CK levels were observed in our cohort.

Several clinically relevant variables were unavailable within the electronic health records. Data regarding physical activity, recent exercise, body composition, and SAMS was not commonly recorded. Given that physical activity is a recognised driver of CK concentrations and that CK elevations can occur irrespective of muscular complaints, these unmeasured factors may have contributed to the residual variability in CK levels (36, 37). Future prospective studies utilising standardised evaluations of exercise habits, muscular complaints, and functional status would yield a more thorough assessment of CK variability with statin treatment.

Furthermore, data on height, body mass index (BMI), body composition, and skeletal muscle mass was unavailable. Despite the inclusion of body weight in the multivariable analysis, it is not sufficient to entirely explain the inter-individual variations in lean muscle mass, a known predictor of CK concentrations (36). Thus, residual confounding associated with body composition cannot be excluded. Future prospective studies utilising standardised anthropometric and body composition parameters are necessary to more accurately define their impact on routine CK variability during statin therapy.

Furthermore, information regarding SAMS, muscle complaints, and statin discontinuation was unavailable. Consequently, the present study evaluates biochemical CK variability rather than clinical statin intolerance, and the findings should not be used to confirm or exclude SAMS in individual patients.

The findings of this study have important implications for clinical practice. CK measurements are frequently obtained during statin therapy, often prompting concern regarding potential muscle toxicity. However, the substantial overlap in CK distributions between statin users and non-users and the absence of a dose-response relationship suggest that modest CK elevations should not automatically be interpreted as evidence of clinically significant SAMS. Instead, CK concentrations should be evaluated alongside clinical symptoms, concurrent medications, and patient-specific characteristics. An individualized approach to CK levels interpretation could support patient-centred therapeutic decision-making regarding statin therapy and promote sustained compliance with evidence-based lipid-lowering treatment in outpatient settings (16, 17).

Findings from the current study should be interpreted within the context of routine biochemical CK level monitoring rather than clinical assessment of SAMS. Although CK is a useful marker of muscle injury, normal CK concentrations do not exclude SAMS, and many patients with clinically relevant muscle symptoms present with normal CK values (11, 38). Accordingly, the present study evaluates factors associated with routine CK variability and should not be interpreted as assessing the incidence or diagnosis of SAMS. Overall, the findings suggest that modest differences in CK concentrations should be interpreted within the broader clinical context rather than in isolation. Patient characteristics, comorbidities, and clinical presentation remain essential when evaluating CK measurements, particularly in asymptomatic patients receiving routine statin therapy. Future prospective studies integrating patient-reported muscle symptoms, treatment discontinuation, and serial CK measurements are needed to better characterize the relationship between CK variability and clinically significant SAMS.

## 5 Conclusion

In this large outpatient cohort, statin use and male sex were independently associated with modest differences in CK concentrations, whereas statin intensity and statin type were not. Importantly, CK levels remained largely within the laboratory reference range, suggesting that the observed differences primarily reflect expected physiological and routine treatment-related variability rather than clinically significant muscle injury in most outpatient statin users. These findings provide real-world evidence to support a balanced interpretation of routine CK measurements during statin therapy and reinforce that laboratory results should be interpreted alongside the patient's clinical presentation and muscle symptoms rather than in isolation.

## Data Availability

The raw data supporting the conclusions of this article will be made available by the authors, without undue reservation.

## References

[1] Almaramihi WS Zahrani M Kamfar R Larry R Alsubaie R. Lipid lowering agent prescription and LDL target achievement in ACS patients in Jeddah, Saudi Arabia. Threshold. (2023) 10:11. 10.24911/IJMDC.51-1685365843

[2] Robert AA Al Dawish MA. Cardiovascular disease among patients with diabetes: the current scenario in Saudi Arabia. Curr Diabetes Rev. (2021) 17:180–5. 10.2174/18756417MTA2kOTQCw 32459609

[3] Adedinsewo D Taka N Agasthi P Sachdeva R Rust G Onwuanyi A. Prevalence and factors associated with statin use among a nationally representative sample of US adults: national health and nutrition examination survey, 2011–2012. Clin Cardiol. (2016) 39:491–6. 10.1002/clc.22577 27505443 PMC5030167

[4] Warden BA Guyton JR Kovacs AC Durham JA Jones LK Dixon DL. Assessment and management of statin-associated muscle symptoms (SAMS): a clinical perspective from the national lipid association. J Clin Lipidol. (2023) 17:19–39. 10.1016/j.jacl.2022.09.001 36115813

[5] Abed W Abujbara M Batieha A Ajlouni K. Statin induced myopathy among patients attending the national center for diabetes, endocrinology, & genetics. Ann Med Surg (Lond). (2022) 74:103304. 10.1016/j.amsu.2022.103304 35145672 PMC8818528

[6] Suliman I Batarfi A Almohammadi H Aljeraisi H Alnaserallah H Alghamdi A. Prevalence of self-reported muscle pain among statin users from national guard hospital, Riyadh. Cureus. (2022) 14:e23463. 10.7759/cureus.23463 35481326 PMC9034880

[7] Bouitbir J Sanvee GM Panajatovic MV Singh F Krähenbühl S. Mechanisms of statin-associated skeletal muscle-associated symptoms. Pharmacol Res. (2020) 154:104201. 10.1016/j.phrs.2019.03.010 30877064

[8] Turner RM Pirmohamed M. Statin-related myotoxicity: a comprehensive review of pharmacokinetic, pharmacogenomic and muscle components. J Clin Med. (2019) 9:22. 10.3390/jcm9010022 31861911 PMC7019839

[9] Kee PS Chin PKL Kennedy MA Maggo SDS. Pharmacogenetics of statin-induced myotoxicity. Front Genet. (2020) 11:575678. 10.3389/fgene.2020.575678 33193687 PMC7596698

[10] Du Souich P Roederer G Dufour R. Myotoxicity of statins: mechanism of action. Pharmacol Ther. (2017) 175:1–16. 10.1016/j.pharmthera.2017.02.029 28223230

[11] Stroes ES Thompson PD Corsini A Vladutiu GD Raal FJ Ray KK. Statin-associated muscle symptoms: impact on statin therapy—European atherosclerosis society consensus panel statement on assessment, aetiology and management. Eur Heart J. (2015) 36:1012–22. 10.1093/eurheartj/ehv043 25694464 PMC4416140

[12] Ismail HM Shora HA Al-Nozha O Yamany HA Al-Nozha F Zaitone SA. Pattern of statin use in patients with diabetes mellitus type 2 for primary and secondary prevention of cardiovascular disease in Saudi Arabia. Afr J Diabetes Med. (2020) 28:20–25.

[13] Divo MJ Martinez CH Mannino DM. Ageing and the epidemiology of multimorbidity. Eur Respir J. (2014) 44:1055–68. 10.1183/09031936.00059814 25142482 PMC4918092

[14] von Elm E Altman DG Egger M Pocock SJ Gøtzsche PC Vandenbroucke JP. The strengthening the reporting of observational studies in epidemiology (STROBE) statement: guidelines for reporting observational studies. Lancet. (2007) 370:1453–7. 10.1016/S0140-6736(07)61602-X 18064739

[15] World Health Organization. Anatomical Therapeutic Chemical (ATC) Classification index with Defined Daily Doses (DDDs). In: Methodology CCfDS, edn. Oslo: World Health Orgnisation (2000).

[16] Heidenreich PA Bozkurt B Aguilar D Allen LA Byun JJ Colvin MM. 2022 AHA/ACC/HFSA guideline for the management of heart failure: a report of the American College of Cardiology/American Heart Association joint committee on clinical practice guidelines. J Am Coll Cardiol. (2022) 79:e263–421. 10.1016/j.jacc.2021.12.012 35379503

[17] Virani SS Newby LK Arnold SV Bittner V Brewer LPC Demeter SH. 2023 AHA/ACC/ACCP/ASPC/NLA/PCNA guideline for the management of patients with chronic coronary disease: a report of the American Heart Association/American College of Cardiology joint committee on clinical practice guidelines. J Am Coll Cardiol. (2023) 82:833–955. 10.1016/j.jacc.2023.04.003 37480922

[18] Lu CY Barratt J Vitry A Roughead E. Charlson and Rx-risk comorbidity indices were predictive of mortality in the Australian health care setting. J Clin Epidemiol. (2011) 64:223–8. 10.1016/j.jclinepi.2010.02.015 21172602

[19] World Health Organisation. International Statistical Classification of Disease and Related Health Problems 10th Revision (ICD-10)-wHO Version for 2016. Geneva: World Health Organization (2016).

[20] Pratt NL Kerr M Barratt JD Kemp-Casey A Kalisch Ellett LM Ramsay E. The validity of the Rx-risk comorbidity index using medicines mapped to the anatomical therapeutic chemical (ATC) classification system. BMJ open. (2018) 8:e021122. 10.1136/bmjopen-2017-021122 29654048 PMC5905736

[21] Alqurain AA Albaharnah M Al Zayer S Ameer L Ghosn S Al-Shaibi S. The prevalence of polypharmacy and hyper-polypharmacy among middle-aged vs. older patients in Saudi Arabia: a cross-sectional study. Front Pharmacol. (2024) 15:1357171. 10.3389/fphar.2024.1357171 38933679 PMC11200110

[22] Cockcroft DW Gault MH. Prediction of creatinine clearance from serum creatinine. Nephron. (1976) 16:31–41. 10.1159/000180580 1244564

[23] Banach M Reiner Ž Surma S Bajraktari G Bielecka-Dabrowa A Bunc M. 2024 Recommendations on the optimal use of lipid-lowering therapy in established atherosclerotic cardiovascular disease and following acute coronary syndromes: a position paper of the international lipid expert panel (ILEP). Drugs. (2024) 84:1541–77. 10.1007/s40265-024-02105-5 39497020 PMC11652584

[24] Gupta R Lodha S Sharma KK Sharma SK Gupta S Asirvatham AJ. Evaluation of statin prescriptions in type 2 diabetes: India heart watch-2. BMJ Open Diabetes Res Care. (2016) 4:e000275. 10.1136/bmjdrc-2016-000275 27648292 PMC5013346

[25] Demoz GT Wahdey S Kasahun GG Hagazy K Kinfe DG Tasew H. Prescribing pattern of statins for primary prevention of cardiovascular diseases in patients with type 2 diabetes: insights from Ethiopia. BMC Res Notes. (2019) 12:1–7. 10.1186/s13104-019-4423-9 31288848 PMC6617647

[26] Zhao M Woodward M Vaartjes I Millett ERC Klipstein-Grobusch K Hyun K. Sex differences in cardiovascular medication prescription in primary care: a systematic review and meta-analysis. J Am Heart Assoc. (2020) 9:e014742. 10.1161/JAHA.119.014742 32431190 PMC7429003

[27] Sadeeqa S Maqsood M Ahmad M. Prevalence of statin induced myopathy in Lahore, Pakistan. Pak J Pharm Sci. (2018) 31:617–622. 29625933

[28] Al-Qurain AA Gebremichael LG Khan MS Williams DB Mackenzie L Phillips C. Prevalence and factors associated with analgesic prescribing in poly-medicated elderly patients. Drugs Aging. (2020) 37:291–300. 10.1007/s40266-019-00742-0 32016823

[29] Brewster LM Mairuhu G Sturk A Van Montfrans GA. Distribution of creatine kinase in the general population: implications for statin therapy. Am Heart J. (2007) 154:655–61. 10.1016/j.ahj.2007.06.008 17892987

[30] Hekimsoy Z Kavalali Oktem I. Serum creatine kinase levels in overt and subclinical hypothyroidism. Endocr Res. (2005) 31:171–5. 10.1080/07435800500371706 16392619

[31] Liu S-R Zhao Z-G Zhai R-R Wang L-J Yang C Ma Q-B. Reversible elevation of creatine kinase and creatinine caused by sintilimab-induced hypothyroidism: a case report. Medicine (Baltimore). (2024) 103:e40080. 10.1097/MD.0000000000040080 39432606 PMC11495781

[32] Fulster S Tacke M Sandek A Ebner N Tschope C Doehner W. Muscle wasting in patients with chronic heart failure: results from the studies investigating co-morbidities aggravating heart failure (SICA-HF). Eur Heart J. (2013) 34:512–9. 10.1093/eurheartj/ehs381 23178647

[33] McDonagh TA Metra M Adamo M Gardner RS Baumbach A Böhm M. ESC Guidelines for the diagnosis and treatment of acute and chronic heart failure. Eur Heart J. (2021) 42:3599–726. 10.1093/eurheartj/ehab368 34922348

[34] Park SW Goodpaster BH Strotmeyer ES De Rekeneire N Harris TB Schwartz AV. Decreased muscle strength and quality in older adults with type 2 diabetes: the health, aging, and body composition study. Diabetes. (2006) 55:1813–8. 10.2337/db05-1183 16731847

[35] DeFronzo RA Tripathy D. Skeletal muscle insulin resistance is the primary defect in type 2 diabetes. Diabetes Care. (2009) 32:S157. 10.2337/dc09-S302 19875544 PMC2811436

[36] Brancaccio P Maffulli N Limongelli FM. Creatine kinase monitoring in sport medicine. Br Med Bull. (2007) 81:209–30. 10.1093/bmb/ldm014 17569697

[37] Baird MF Graham SM Baker JS Bickerstaff GF. Creatine-kinase-and exercise-related muscle damage implications for muscle performance and recovery. J Nutr Metab. (2012) 2012:960363. 10.1155/2012/960363 22288008 PMC3263635

[38] Newman CB Preiss D Tobert JA Jacobson TA Page RL Goldstein LB. Statin safety and associated adverse events: a scientific statement from the American Heart Association. Arterioscler Thromb Vasc Biol. (2019) 39:e38–81. 10.1161/ATV.0000000000000073 30580575

