# Supplementary material for: Factors associated with creatine kinase variability among statin users in routine outpatient care: a retrospective cross-sectional study
Source: Front Cardiovasc Med. 2026 Aug 28;13:1903467. doi: 10.3389/fcvm.2026.1903467 (PMC13561765; doi:10.3389/fcvm.2026.1903467)
Supplement: Supplementary file 1 [file Datasheet1.docx]

**Determinants of Creatine Kinase Variability Among Statin Users in Routine Outpatient Care: A Retrospective Cross-Sectional Study**

**Supplementary Materials**

Table S1. Distribution of creatine kinase levels among study participants

| **Percentile** | **CK (IU/L)** |
| --- | --- |
| 1st | 29 |
| 5th | 39 |
| 25th | 68 |
| 50th (Median) | 94 |
| 75th | 121 |
| 95th | 157 |
| 99th | 175 |

*CK, creatine kinase. Values represent the distribution of creatine kinase measurements among all study participants (n = 6,517)*

Table S2. Characteristics according to creatine kinase quartiles

| Characteristic | Q1 | Q2 | Q3 | Q4 |
| --- | --- | --- | --- | --- |
| n | 1630 | 1629 | 1629 | 1629 |
| Age, years | 52 | 52 | 52 | 52 |
| Male, % | 36.7 | 46.3 | 47.1 | 62.4 |
| Weight, kg | 70 | 70 | 70 | 71 |
| CrCl, mL/min | 90.1 | 91.1 | 88.2 | 89.9 |
| Statin users, % | 23.1 | 46.7 | 45.5 | 82.6 |
| NPM | 5 | 5 | 5 | 5 |
| CCI | 2 | 2 | 2 | 2 |

*n = number of patients, kg = Kilogram, CrCl = Creatinine clearance, NPM = Number of prescribed medications, CCI = Charlson comorbidity index.*

Table S3. Characteristics of the cohort classified based on diabetes status.

| Characteristics | Entire cohort | Diabetic patients | Non-diabetic patients | *p*-value |
| --- | --- | --- | --- | --- |
|  | *n = 6517* | *n = 2275* | *n = 4242* |  |
| Age (year), median (IQR) | 52 (45 – 63) | 52 (45 – 62) | 52 (45 – 63) | 0.9 |
| Gender (Female sex), n (%) | 3380 (52) | 1198 (52.7) | 2182 (51.4) | 0.4 |
| CCI, median (IQR) | 2 (1 – 3) | 2 (1 – 3) | 2 (1 – 3) | 0.4 |
| NPM, median (IQR) | 5 (2 – 10) | 5 (2 – 11) | 5 (2 – 9) | 0.07 |
| CrCl (mL/min), mean (SD) | 96 (46) | 94 (48) | 98 (44) | < 0.001 |
| Body Weight (Kg), median (IQR) | 70 (63 – 80) | 70 (61 – 80) | 70 (65 – 80) | 0.7 |
| Creatinine Kinase (IU/L), mean (SD) | 96 (36) | 104 (35) | 91 (35) | < 0.001 |
| Statin use, n (%) | 3223 (49.5) | 1798 (79) | 1425 (33.6) | < 0.001 |
| Comorbidities | | | | |
| Liver disease, n (%) | 340 (5) | 147 (6.5) | 193 (4.5) | 0.001 |
| Renal disease, n (%) | 205 (3) | 92 (4) | 113 (2.7) | 0.002 |
| Anemia, n (%) | 2163 (33) | 837 (36.8) | 1326 (31.3) | < 0.001 |
| Heart Failure, n (%) | 3072 (47) | 1513 (66.5) | 1559 (36.8) | < 0.001 |
| Hypertension, n (%) | 3168 (39) | 1619 (71.2) | 1549 (36.5) | < 0.001 |
| Ischemic Heart Disease, n (%) | 3503 (54) | 1684 (74) | 1819 (42.9) | < 0.001 |
| Thyroid disorder, n (%) | 839 (13) | 336 (14.8) | 503 (11.9) | 0.001 |

Table S4. Characteristics of the cohort classified based on cardiovascular disease burden.

| Characteristics | Entire cohort | Cardiovascular diseases patients | Non-cardiovascular disease patients | *p*-value |
| --- | --- | --- | --- | --- |
|  | *n = 6517* | *n = 3797* | *n = 2720* |  |
| Age (year), median (IQR) | 52 (45 – 63) | 52 (45 – 62) | 52 (45 – 63) | 0.2 |
| Gender (Female sex), n (%) | 3380 (52) | 1960 (51.6) | 1420 (52.2) | 0.6 |
| CCI, median (IQR) | 2 (1 – 3) | 2 (1 – 3) | 2 (1 – 3) | 0.2 |
| NPM, median (IQR) | 5 (2 – 10) | 5 (3 – 10) | 4 (2 – 9) | < 0.001 |
| CrCl (mL/min), mean (SD) | 96 (46) | 93 (46) | 101 (45) | < 0.001 |
| Body Weight (Kg), median (IQR) | 70 (63 – 80) | 70 (63 – 80) | 70 (64 – 80) | 0.3 |
| Creatinine Kinase (IU/L), mean (SD) | 96 (36) | 102 (36) | 87 (34) | < 0.001 |
| Statin use, n (%) | 3223 (49.5) | 2605 (68.6) | 618 (22.7) | < 0.001 |
| Comorbidities | | | | |
| Diabetes mellitus, n (%) | 2275 (34.9) | 1757 (46.3) | 518 (19) | < 0.001 |
| Liver disease, n (%) | 340 (5) | 251 (6.6) | 89 (3.3) | < 0.001 |
| Renal disease, n (%) | 205 (3) | 152 (4) | 53 (1.9) | < 0.001 |
| Anemia, n (%) | 2163 (33) | 1171 (30.8) | 992 (36.5) | < 0.001 |
| Heart Failure, n (%) | 3072 (47) | 3072 (80.9) | 0 (0) | < 0.001 |
| Hypertension, n (%) | 3168 (39) | 3168 (83.4) | 0 (0) | < 0.001 |
| Ischemic Heart Disease, n (%) | 3503 (54) | 3503 (92.3) | 0 (0) | < 0.001 |
| Thyroid disorder, n (%) | 839 (13) | 401 (10.6) | 438 (16.1) | < 0.001 |

Table S5. Characteristics of the cohort classified based on anemia status.

| Characteristics | Entire cohort | Anemia patients | Non-anemia patients | *p*-value |
| --- | --- | --- | --- | --- |
|  | *n = 6517* | *n = 2163* | *n = 4354* |  |
| Age (year), median (IQR) | 52 (45 – 63) | 52 (44 – 63) | 52 (45 – 62) | 0.9 |
| Gender (Female sex), n (%) | 3380 (52) | 1152 (53.3) | 2228 (51.2) | 0.1 |
| CCI, median (IQR) | 2 (1 – 3) | 2 (1 – 3) | 2 (1 – 3) | 0.8 |
| NPM, median (IQR) | 5 (2 – 10) | 4 (1 – 3) | 5 (3 – 10) | < 0.001 |
| CrCl (mL/min), mean (SD) | 96 (46) | 98 (52) | 96 (43) | 0.06 |
| Body Weight (Kg), median (IQR) | 70 (63 – 80) | 70 (60 – 80) | 70 (65 – 80) | 0.009 |
| Creatinine Kinase (IU/L), mean (SD) | 96 (36) | 94 (35) | 96 (36) | 0.01 |
| Statin use, n (%) | 3223 (49.5) | 998 (46.1) | 2225 (51.1) | < 0.001 |
| Comorbidities | | | | |
| Diabetes mellitus, n (%) | 2275 (34.9) | 837 (38.7) | 1438 (33) | < 0.001 |
| Liver disease, n (%) | 340 (5) | 168 (7.8) | 172 (4) | < 0.001 |
| Renal disease, n (%) | 205 (3) | 153 (7.1) | 52 (1.2) | < 0.001 |
| Heart Failure, n (%) | 3072 (47) | 933 (43.1) | 2139 (49.1) | < 0.001 |
| Hypertension, n (%) | 3168 (39) | 1001 (46.3) | 2167 (49.8) | 0.008 |
| Ischemic Heart Disease, n (%) | 3503 (54) | 1089 (50.3) | 2414 (55.4) | < 0.001 |
| Thyroid disorder, n (%) | 839 (13) | 295 (13.6) | 544 (12.5) | 0.2 |

Table S6. Multivariable Models Evaluating Factors Associated with Creatine Kinase Levels

| Variable | Model 1 Patient Factors β (p) | Model 2 + Statin Use β (p) | Model 3 Fully Adjusted β (p) | Model 4 Statin Users Only β (p) |
| --- | --- | --- | --- | --- |
| Age | -0.096 (0.010) | -0.010 (0.752) | -0.010 (0.770) | -0.016 (0.753) |
| Female sex | -14.832 (<0.001) | -13.980 (<0.001) | -13.946 (<0.001) | -12.978 (<0.001) |
| Weight | 0.141 (<0.001) | 0.042 (0.108) | 0.040 (0.134) | 0.056 (0.141) |
| CrCl | -0.082 (<0.001) | -0.014 (0.235) | -0.013 (0.285) | -0.017 (0.324) |
| Statin use | — | 31.695 (<0.001) | 31.836 (<0.001) | — |
| Diabetes mellitus | — | — | 1.036 (0.257) | 1.292 (0.281) |
| Hypertension | — | — | -1.120 (0.494) | -1.397 (0.551) |
| Ischaemic heart disease | — | — | -0.564 (0.738) | -2.801 (0.286) |
| Heart failure | — | — | 1.056 (0.365) | 2.171 (0.212) |
| Thyroid disorder | — | — | 2.600 (0.026) | 3.337 (0.057) |
| Renal disease | — | — | 1.117 (0.625) | — |
| Liver disease | — | — | -0.837 (0.633) | — |
| Atorvastatin* | — | — | — | 4.766 (0.315) |
| Rosuvastatin* | — | — | — | 3.905 (0.442) |
| Statin intensity* | — | — | — | 0.591 (0.675) |
| Adjusted R² | 0.042 | 0.236 | 0.236 | 0.037 |

*Model 1: Age, sex, weight, and creatinine clearance.*

*Model 2: Model 1 plus statin exposure.*

*Model 3: Model 2 plus diabetes mellitus, hypertension, ischaemic heart disease, heart failure, thyroid disorder, renal disease, and liver disease.*

*Model 4: Restricted to statin users and additionally adjusted for statin intensity and statin type.*

** Reference statin = simvastatin.*

*CrCl = creatinine clearance.*

*β coefficients represent adjusted differences in CK levels (IU/L).*

Table S7: Biochemistry laboratory test results are classified based on statin administration and dosage intensity.

|  | Whole cohort | Statin users | Statin non-users | *p*-value * | Low dose | Medium dose | High dose | *p*-value ǂ |
| --- | --- | --- | --- | --- | --- | --- | --- | --- |
|  | *n = 6517* | *n = 3223* | *n = 3294* |  | *n =55* | *n = 1700* | *n = 1468* |  |
| Vitamin D3 (nmol/L), mean (SD) | 117 (55) | 117 (55) | 118 (55) | 0.7 | 118 (52) | 118 (55) | 116 (56) | 0.6 |
| Albumin (g/L), mean (SD) | 40 (9) | 40 (9) | 40 (9) | 0.5 | 42 (8) | 40 (9) | 40 (9) | 0.1 |
| Alanine Aminotransferase (IU/L), mean (SD) | 32 (33) | 32 (15) | 33 (44) | 0.04 | 33 (15) | 32 (15) | 32 (14) | 0.6 |
| Aspartate Aminotransferase (IU/L), mean (SD) | 21 (11) | 20 (12) | 23 (8) | 0.07 | 20 (11) | 20 (11) | 20 (12) | 0.9 |
| Cholesterol (mmol/L), mean (SD) | 3.6 (1.3) | 3.6 (1.3) | 3.6 (1.3) | 0.6 | 3.7 (1.2) | 3.7 (1.3) | 3.6 (1.4) | 0.8 |
| Low-density lipoprotein (LDL) (mmol/L), mean (SD) | 2.3 (1.2) | 2.4 (1.2) | 2.3 (1.2) | 0.2 | 2.1 (1) | 2.4 (1.2) | 2.3 (1.2) | 0.2 |
| High-density lipoprotein (HDL) (mmol/L), mean (SD) | 1.6 (0.7) | 1.6 (0.7) | 1.6 (0.7) | 0.8 | 1.6 (0.8) | 1.6 (0.7) | 1.6 (0.7) | 0.2 |
| Triglyceride (mmol/L), mean (SD) | 2.4 (1.6) | 2.4 (1.6) | 2.4 (1.6) | 0.6 | 2.4 (1.8) | 2.4 (1.6) | 2.4 (1.6) | 0.9 |
| Creatine kinase (IU/L), mean (SD) | 96 (36) | 112 (34) | 80 (30) | < 0.001 | 110 (34) | 112 (34) | 115 (34) | 0.3 |
| Gamma-Glutamyl Transpeptidase (IU/L), mean (SD) | 14.8 (30) | 15.3 (32) | 14.2 (30) | 0.3 | 15 (20) | 16 (37) | 14 (26) | 0.9 |
| C-reactive Protein, mean (SD) | 3.8 (3.6) | 3.8 (3.8) | 3.7 (3.5) | 0.1 | 2.9 (2) | 3.8 (3.7) | 3.9 (3.9) | 0.4 |

*IQR = Interquartile range, SD = Standard deviation, n = number of participants, CCI = Charlson comorbidity index, NPM = Number of prescribed medications, CrCl = Creatinine clearance. * p-values were generated using independent t-tests or Mann–Whitney U tests, as appropriate. ǂ p-values were generated using ANOVA or Kruskal–Wallis tests, as appropriate. Laboratory parameters were analyzed using available laboratory records from routine clinical testing.*
